# Prospective Analysis of Multidisciplinary (MDT)-Based Cross-Sectional Imaging to Predict the Histology of Soft Tissue Tumors (BACH-Trial)

**DOI:** 10.3390/cancers18050784

**Published:** 2026-02-28

**Authors:** Katja Fechner, Henriette Golcher, Maximilian Brunner, Norbert Meidenbauer, Sabine Semrau, Michael Uder, Georg F. Weber, Axel Denz, Abbas Agaimy, Robert Grützmann

**Affiliations:** 1 Department of Surgery, Friedrich-Alexander-University Erlangen-Nuremberg (FAU), Krankenhausstraße 12, 91054 Erlangen, Germany; henriette.golcher@uk-erlangen.de (H.G.); maximilian.brunner@uk-erlangen.de (M.B.); georg.weber@uk-erlangen.de (G.F.W.); axel.denz@uk-erlangen.de (A.D.); robert.gruetzmann@uk-erlangen.de (R.G.); 2 Department of Internal Medicine 5, Hematology and Oncology, Friedrich-Alexander-University Erlangen-Nuremberg (FAU), Ulmenweg 18, 91054 Erlangen, Germany; norbert.meidenbauer@uk-erlangen.de; 3 Department of Radiation Oncology, Friedrich-Alexander-University Erlangen-Nuremberg (FAU), Universitätsstraße 27, 91054 Erlangen, Germany; sabine.semrau@uk-erlangen.de; 4 Department of Radiology, Friedrich-Alexander-University Erlangen-Nuremberg (FAU), Ulmenweg 18, 91054 Erlangen, Germany; michael.uder@uk-erlangen.de; 5 Institut of Pathology, Friedrich-Alexander-University Erlangen-Nuremberg (FAU), Krankenhausstraße 8-10, 91054 Erlangen, Germany; abbas.agaimy@uk-erlangen.de; 6 Comprehensive Cancer Center Erlangen-EMN (CCC ER-EMN), 91054 Erlangen, Germany; 7 Comprehensive Cancer Center Alliance WERA (CCC WERA), 91054 Erlangen, Germany; 8 Bavarian Cancer Research Center (BZKF), 91052 Erlangen, Germany

**Keywords:** soft tissue tumors, prospective analysis, imaging, pathology, multidisciplinary sarcoma board

## Abstract

**Background**: The necessity of a pre-therapeutic biopsy for soft tissue tumors is assessed differently depending on imaging. We examined the concordance of histopathological and radiological imaging-based diagnoses of soft tissue tumors in a monocentric, multidisciplinary sarcoma board. **Methods**: From October 2022 to December 2024, we prospectively included 184 patients presenting with preoperative imaging but without prior histology who are presented at the multidisciplinary sarcoma board of the University Hospital of Erlangen. We evaluated tumor dignity (benign/malignant) and most probable tumor subtype based on cross-sectional imaging assisted by the demographic and anatomic characteristics of individual cases. This assessment was then compared with the final pathological results. **Results**: We classified 75 tumors as benign and 109 tumors as malignant. Of the 75 patients with a suspected benign tumor, 66 (88%) had a benign diagnosis confirmed by pathological assessment, while two (2.7%) had a malignant tumor and seven (9.3%) an intermediate biology tumor. Of the 109 patients with suspected malignant tumors, 69 (63.3%) had a malignant pathology, while 30 (27.5%) had a benign pathology, and 10 (9.2%) an intermediate tumor. Matching the multidisciplinary sarcoma board’s assessment with the pathological results revealed significant sensitivity and a negative predictive value for malignant tumors, as well as a significant positive predictive value and specificity for benign tumors. **Conclusions**: The study shows that, despite the high degree of predictability at an experienced sarcoma center, imaging cannot completely replace biopsies and caution should be exercised when deciding against a biopsy. It is emphasized that the decision not to perform a biopsy can only be made in cases where lipomatous tumors appear benign in imaging procedures, and only in an experienced center.

## Simple Summary

Soft tissue tumors are rare and heterogeneous in imaging, histology, and prognosis. The prospective study addresses the clinically relevant problems of whether a biopsy of a benign tumor can be safely omitted and whether the exact tumor type can also be predicted based on radiological and clinical/anatomical criteria. The aim of our prospective study is therefore to examine the concordance between histopathological and radiological imaging-based diagnoses of soft tissue tumors in a monocentric, multidisciplinary sarcoma board. We were able to show that in 184 included patients, the comparison of the multidisciplinary sarcoma board’s assessment with the pathological results revealed significant sensitivity and negative predictive value for malignant tumors, as well as significant positive predictive value and significant specificity for benign tumors. The study also shows that, despite the high predictability in an experienced sarcoma center, imaging cannot completely replace a biopsy, and caution should be exercised when deciding against a biopsy.

## 1. Introduction

Soft tissue tumors are rare and heterogeneous in imaging, histology and prognosis with a frequency of benign versus malignant (sarcomas) of approximately 100:1 in unbiased epidemiological data. There are more than 80 different subtypes of soft tissue tumors that can occur anywhere in the body and are often managed with multimodal treatment approaches [1,2]. The preferred primary imaging modalities for tumor localization are contrast-enhanced magnetic resonance imaging (MRI) of the extremities and computed tomography (CT) of the abdomen and retroperitoneum. Following an appropriate imaging assessment, the standard diagnostic approach consists of biopsies and histopathological evaluation [2]. For lesions smaller than 3 cm, an excisional biopsy is the preferred option [2,3,4]. It should be noted that intratumoral heterogeneity exists in some sarcomas [5,6]. This leads to differences in pathology accuracy in biopsies and resected specimens, particularly regarding the tumor grade [7]. Studies have correlated imaging and pathology in rare mesenchymal tumors of the pelvis, uterine sarcomas, imaging for predicting tumor grading, and assessing the treatment response in sarcomas [6,8,9,10,11,12,13]. In addition, pre-therapeutic imaging is important for therapy planning, i.e., whether neoadjuvant therapy is necessary, and also for surgical planning. Incomplete resection should be avoided in malignant tumors, as this can lead to a poorer prognosis. In summary, pre-treatment assessment contributes to joint decision-making, and survival rates improve when specialized multidisciplinary committees for sarcoma patients are used in sarcoma reference centers [2,14]. We present a prospective study correlating the radiological assessment of soft tissue tumors with pathological results in a multidisciplinary sarcoma board setting. In a second analysis, we correlated the pathological results of pre-treatment biopsies with those of resected specimens. This study addresses the clinically relevant question of whether a biopsy of a tumor deemed benign based on radiological and clinical criteria can safely be omitted. In addition, the area of interest in heterogeneous-appearing and biphasic tumors is discussed and determined in the MDT setting. This always helps to avoid non-diagnostic necrotic biopsies and to facilitate the highest grade in heterogeneous tumors that are likely going to receive preoperative treatment.

## 2. Materials and Methods

In this prospective study, we identified 297 patients (>18 years) who were presented with cross-sectional imaging but without histological diagnosis at the multidisciplinary sarcoma board of the University Hospital of Erlangen from October 2022 to December 2024. We excluded 113 patients due to a lack of histology, bone tumors, and tumors of the spleen. The remaining 184 patients were included in the final analysis. An oncologist, a surgical oncologist, a sarcoma pathologist, a radiation oncologist, and a radiologist participated in the sarcoma board. All participants actively took part in the assessment, which was based on a majority principle. In addition, all participants have more than 10 years of experience in the diagnosis and treatment of soft tissue tumors. We assessed tumor type (benign or malignant) and, if possible, the exact tumor subtype based on cross-sectional imaging supplemented by the clinical–anatomic context (age of patient, site of tumor, depth of tumor). There were no predefined imaging criteria. Blinding was not necessary or even possible, as no pathological results were available at the time of the assessment.

**Figure 1:**
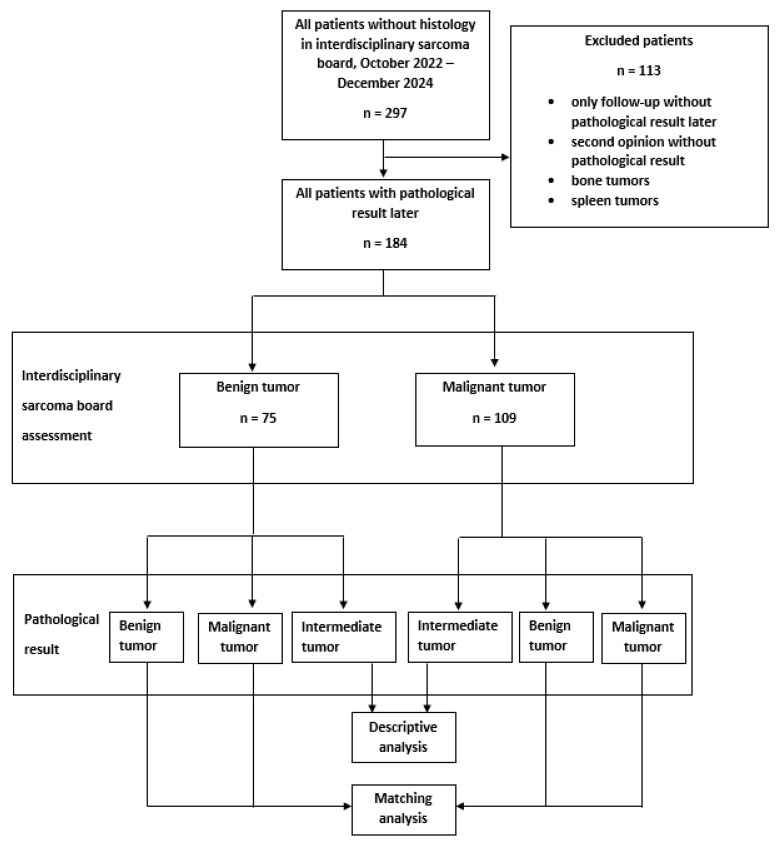
Flowchart of study.

This assessment was then compared with the pathological results of biopsies and/or resected tumor specimens (Figure 1). As tumors in the intermediate biology category cannot be predicted from imaging (with few exceptions such as desmoid and tenosynovial giant cell tumors), imaging prediction was for benign vs. malignant in most cases only. The biopsies were predominantly performed as a CT-guided core-needle biopsy by experienced radiologists. In all cases involving larger lipomatous tumors, an MDM2 analysis was performed.

A matching analysis was performed for pathological benign and malignant tumors. Furthermore, we analyzed the patients’ demographic characteristics (e.g., age and gender), the anatomical localization of the tumors within the body (e.g., extremity, intra-abdominal/retroperitoneal, trunk, or head/neck), imaging methods (e.g., MRI, CT or both) and the grading. If possible, a suspected diagnosis for the tumor was also made as part of the MDT assessment.

Pathological grading was differentiated into five categories: first, malignant tumor and no sarcoma; second, G1 sarcoma; third, non-G1 sarcoma; fourth, no existing grading in sarcoma; and fifth, benign tumors.

Desmoid tumors, tenosynovial giant cell tumors (pigmented villonodular synovitis = PVNS), solitary fibrous tumors and superficial CD34-positive fibroblastic tumors (SCPFT) are classified as intermediate tumors and were analyzed descriptively. Intermediate tumors were excluded from further analysis, as the MDT assessment only classified tumors as benign or malignant. The intermediate tumors would therefore all be classified “incorrectly”. However, as this tumor category occurs in practice and overlaps with benign and malignant tumors in diagnostic terms, we have included it in the study as a descriptive analysis.

In addition, when performing a biopsy and surgery, we correlated the pathological results of the biopsies with the resected tumors. The characteristics of all the patients included can be found in Table S1 of the Supplementary Material.

Statistical data analysis was performed using SPSS Statistics (version 28, IBM Corp., Armonk, NY, USA). We calculated comparisons of ordinal and metric data using the Mann–Whitney U test. For categorial data, the chi-square test was used. Statistical significance was set at *p* < 0.05.

This study was approved by the Ethics Committee of Friedrich-Alexander-University Erlangen-Nuremberg (ethical code number 21-211-Br).

## 3. Results

From October 2022 to December 2024, 297 patients without histology were presented to the interdisciplinary sarcoma board. Of those, 184 patients (94 female) were included in this study. The median age was 58.5 years, ranging from 21 to 90 years. Of the cases, 143 (77.7%) had an MRI, 31 (16.8%) had a CT, and 10 (5.4%) had both an MRI and a CT. We analyzed the characteristics of all patients and of the patients with a benign, malignant and intermediate tumor in the pathological results (Table 1).

The malignant tumor group was significantly older than the other groups (*p* < 0.001). There was no significant difference in terms of gender between the three tumor groups. However, there was a tendency for slightly more women to be present in the benign and intermediate tumor groups, and for more male patients to be affected in the malignant tumor group.

There were significant differences in tumor localization (*p* < 0.001) with benign and malignant tumors frequently occurring in the extremities, and intermediate tumors most commonly occurring on the trunk. All tumors were diagnosed significantly more frequently by MRI (*p* < 0.001).

### 3.1. Multidisciplinary Sarcoma Board Assessment

In the multidisciplinary assessment, we classified 75 tumors as benign and 109 as malignant. Of the 75 patients with a benign tumor, 66 (88%) had a benign diagnosis confirmed by pathological findings, while two (2.7%) had a malignant tumor (pleomorphic liposarcoma and G1 liposarcoma) and seven (9.3%) had an intermediate tumor. The 66 benign tumors were 44 lipomas, eight Schwannomas, two epidermal cysts, two hematomas, and one each of the following: splenoma/splenosis, fat tissue necrosis, myxoma, myxoma with associated lipoma, vascular malformation, neurofibroma, gouty tophus, lymphangioma, chronic inflammation and lymph node.

Of the 66 cases with benign tumors, 63 patients (95.5%) received, in the assessment by MDT, a suspected diagnosis based on imaging techniques. In 55 of these patients (87.3%), the correct tumor subtype was predicted in the MDT assessment.

Of the 109 patients with suspected malignant tumors, 69 (63.3%) had a malignant pathology, while 30 (27.5%) had a benign pathology and 10 (19.2%) an intermediate tumor.

Of the 30 cases with a benign pathology, 15 were initially suspected to be liposarcoma but were found to be lipoma upon a final pathology. Of the 69 cases involving a malignant pathology, a suspected diagnosis by MDT assessment was made in 56 patients (81.2%) based on imaging. The correct tumor subtype was identified in 33 of these cases (58.9%): nine undifferentiated pleomorphic sarcomas, nine liposarcomas, six lymphomas, five gastrointestinal stromal tumors, two myxofibrosarcomas, one neuroendocrine carcinoma metastasis and one lung cancer metastasis.

### 3.2. Matching Analysis: Benign and Malignant Tumors in Pathological Results

There was a significant correlation between MDT assessments and pathological results (*p* < 0.001) for benign and malignant tumors (Table 2).

The sensitivity for malignancy was 97.2%, and the specificity was 68.8%. The positive predictive value for malignancy was 69.7%, and the negative predictive value was 97.1%. In terms of benignity, sensitivity was 68.8%, specificity was 97.2%, positive predictive value 97.1%, and negative predictive value was 69.7%.

In the next step, we analyzed the patients’ characteristics in more detail according to correct and incorrect matching (Table 3).

When considering all pathologically benign and malignant tumors, there were significantly more benign tumors with incorrect matching (grading, *p* < 0.001). In addition, in pathological malignant evaluation by the tumor board, the correct matching with pathological malignancy was significantly better with CT. However, no significant differences were found in the other characteristics, including age, gender or localization, in the multidisciplinary sarcoma board.

The two cases that were classified as benign in the MDT but were pathologically malignant represent an important patient group. The first patient had a pleomorphic liposarcoma that was 5 cm in size and located subcutaneously on the trunk. Due to its subcutaneous location, size of 5 cm, and assessment as benign by the MDT, we decided against a biopsy but nevertheless performed an extensive resection, which was pathologically confirmed as an R0 resection. The procedure was oncologically correct in this case, but could have worsened the prognosis in the case of marginal resection, or R1/R2 resection.

The second patient had a G1 liposarcoma on the thigh measuring 16 cm on MRI. We assessed the tumor as benign but nevertheless performed a CT-guided biopsy due to the size of the tumor and its deep intramuscular location. The marginal resection was performed correctly from an oncological perspective and resulted in an R0 situation. An incorrect resection would have increased the risk of local recurrence.

### 3.3. Intermediate Tumors

The 17 intermediate tumors in the pathological results were eight desmoid tumors, four solitary fibrous tumors, two superficial CD34-positive fibroblastic tumors (SCPFT), two tenosynovial giant cell tumors, and one atypical pleomorphic/spindle cell lipomatous tumor (AP/SLT). Of these, seven were previously assessed as benign and ten as malignant by the multidisciplinary tumor board.

### 3.4. Pathological Results of Biopsies and Resected Tumors

Of the 71 patients with histopathologically verified malignant tumors, 34 (47.9%) underwent a biopsy and resection, with concordant histology in 31 (91.2%) cases. In three cases, there were minor, not significant, variations between the biopsy diagnosis and resection: a melanoma in the biopsy with a complete response after therapy in the resected specimen; a myxofibrosarcoma in the biopsy; and a dedifferentiated liposarcoma with a myxofibrosarcoma-like dedifferentiated morphology in the resection. The third case was judged as inclusive for lipoma vs. G1 liposarcoma in the biopsy: the resection was consistent with liposarcoma and contained a focal dedifferentiated component.

Among all 96 patients with benign tumors, a pathological result was available for the biopsy and resected specimen in 31 patients (32.3%), and the results were identical in 25 patients (80.6%). In six cases (19.4%), the biopsy was inconclusive, making it unclear whether the tumor was a lipoma or a G1 liposarcoma or a myxoma. Pathological results from biopsies and final resection specimens were available for five patients with 17 pathologically intermediate tumors. These results were consistent, including three solitary fibrous tumors and two desmoid tumors.

## 4. Discussion

A pre-therapeutic assessment is crucial for shared decision-making in sarcoma boards at sarcoma reference centers [2,4,13].

This study is a prospective, monocentric analysis of cross-sectional imaging used to predict the histology of soft tissue tumors. The aim is to verify the accuracy of predictions regarding whether tumors are benign or malignant, thereby reducing the need for unnecessary biopsies.

Only tumors that had been assessed by the tumor board without initial histology were included, thereby excluding patients who had already received an external diagnosis. This may lead to a distortion of the epidemiology from a methodological perspective.

In our study, there were significant differences in tumor localization (*p* < 0.001) with benign and malignant tumors frequently occurring in the extremities, and intermediate tumors most commonly occurring on the trunk. According to the literature, soft tissue sarcomas most frequently occur in the extremities [15,16,17]. The intermediate tumors were significantly more prevalent in the trunk, primarily due to the fact that the majority of tumors were desmoid tumors located in the trunk/abdominal wall which occur most frequently in this location [18,19].

In accordance with the literature, the malignant tumor group was significantly older than the other groups (*p* < 0.001) [4,20]. There was no significant difference in terms of gender between the three tumor groups. However, there was a tendency for slightly more women to be present in the benign and intermediate tumor groups, and for more male patients to be affected in the malignant tumor group. Advances in imaging technology have also revolutionized the diagnosis of soft tissue tumors [21]. Contrast-enhanced magnetic resonance imaging (MRI) of the extremities and computed tomography (CT) for abdominal and retroperitoneal tumor localization are the preferred primary imaging modalities [2,21,22]. In our study, MRI was the main imaging modality for all tumors (*p* < 0.001). In our study, the assessment was always based on the imaging that was available, and no further imaging was requested.

Studies have correlated imaging and pathology in rare mesenchymal tumors of the pelvis and uterine sarcomas. Imaging has also been used to predict tumor grading and assess the treatment response in sarcomas [6,8,9,10,11,12,23]. In our study, a team of sarcoma experts within the interdisciplinary sarcoma board evaluated benign or malignant tumors based on additional details available to the board, such as the patient’s age at initial diagnosis, tumor localization, and tumor size based on imaging. The board also considered imaging characteristics of the tumor, such as homogeneity or inhomogeneity, the presence of fatty tumor components, and limited growth or infiltration into neighboring organs.

Therefore, our study is based on real-life data and does not involve any strictly defined criteria.

As the multidisciplinary assessment predicted both benign and malignant tumors, but the pathological findings also included intermediate tumors, the latter were excluded from the matching analysis and analyzed descriptively. The matching analysis of imaging and pathological findings for benign and malignant tumors showed that we significantly assessed pathologically malignant tumors as malignant, with a sensitivity of 97.2% and a high negative predictive value of 97.1%. In such cases, a biopsy should be performed to allow a decision to be made regarding possible neoadjuvant therapy and the extent of surgery in terms of oncological resection. Overall, however, the prediction for malignant tumors was lower in the assessment (positive predictive value 69.7%), as many pathologically benign tumors were included. Nevertheless, a biopsy should be performed if malignancy is suspected in the assessment.

Patients with a benign tumor that is incorrectly classified as malignant are generally at risk of being overtreated. Upon closer examination, half of this group was found to have G1 liposarcoma, which was found to be a lipoma upon pathological examination. Distinguishing between G1 liposarcoma and lipoma using imaging techniques can be difficult, even with pathological examination by a biopsy. If imaging and the biopsy could reliably diagnose lipoma, these cases could be clinically monitored without surgery in asymptomatic patients with tumors of a constant size. In these cases where the findings are unclear (G1 liposarcoma or lipoma), our clinic performs marginal resection to ensure that the correct oncological surgery is performed if the final pathology reveals a G1 liposarcoma.

Surprisingly, the matching analysis also showed that multidisciplinary assessments correctly predicted benign tumors in 97.1% of cases (positive predictive value). There was also high specificity, at 97.2%. In these cases, a biopsy could be omitted and primary surgery performed if indicated due to symptoms or size. The two pathologically malignant tumors that were incorrectly classified as benign in the interdisciplinary assessment were a G1 liposarcoma and a pleomorphic liposarcoma. Primary surgery was performed on these tumors, meaning that neoadjuvant therapy was not necessary and a pre-therapeutic biopsy could be avoided. In the case of pleomorphic liposarcoma, a wide resection was correctly performed as an R0 resection in the subcutaneous location, whereby a marginal resection would not have been sufficient. This shows that caution is still required and that imaging does not completely replace histological confirmation.

MRI is the imaging technique of choice for the characterization and local staging of musculoskeletal soft tissue masses [4,21,24,25].

According to the literature, in some cases, the imaging features of benign and malignant soft tissue tumors overlap; therefore, pathological analysis is necessary for a definitive diagnosis [13,26,27,28,29]. Crombé et al. described an example that a well-defined and homogeneous soft-tissue myxoid tumor can correspond to a myxoma, a myxoid/round cells liposarcoma, a low-grade fibromyxoid chondrosarcoma, or a small extraskeletal myxoid chondrosarcoma [13]. Furthermore, this overlap was observed in tumors with dense vascularization, such as hematomas, neurofibromas, and nodular fasciitis [28,30,31].

Key features in the clinical presentation and MRI findings enable radiologists to diagnose tumors and narrow the differential diagnosis to specific pathological entities [11]. The MRI features—a large size, deep localization, peritumoral enhancement, heterogeneous signal intensity of 50% on T2-weighted imaging, ill-defined margins, and the presence of necrotic tumor areas—were associated with high-grade sarcomas [5,32,33]. In addition, some specific subtypes of sarcomas exhibit particular MRI characteristics, such as the “tail sign” in undifferentiated pleomorphic sarcoma and myxofibrosarcoma, the “water-like” appearance in myxofibrosarcomas, or the “triple sign” in synovial sarcomas [33].

Furthermore, to the assessment of the tumor location, anatomical relationships and dimension, MRI enables us to depict the tumor content. In particular, the diagnostic orientation can be facilitated by the identification of myxoid stroma, fatty content, necrotic content, hemorrhagic content, cystic content, fibrotic content, and their combination [13].

Therefore, in our diagnostic considerations, the clinical presentation, the age of the patient, and the tumor location (e.g., tumor size, infiltration, and heterogeneity) are also crucial for predicting a malignant tumor and making the correct diagnosis.

In summary, the data in the literature report conflicting results on the diagnostic accuracy of MRI in identifying the lesion histotype, with results varying from 25% to 90% [34,35,36].

**Figure 2:**
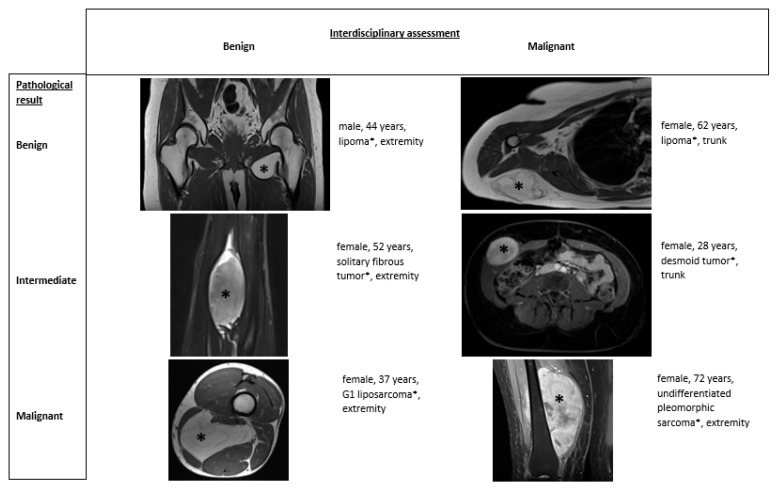
Different tumor types with multidisciplinary assessment and pathological result.

Figure 2 shows examples of different tumor types and the correlation between multidisciplinary assessment and pathological results.

In our study, CT was most frequently used to assess tumors in the intra-abdominal and retroperitoneal areas. Here, it was significant that CT is better at predicting malignant findings in this area.

This is consistent with other studies that have already shown that contrast-enhanced computed tomography (CT) is the most useful and widely used primary imaging method for retroperitoneal tumors [36,37].

It should be noted that intratumoral heterogeneity exists in some sarcomas [5,6]. This leads to differences in pathology accuracy between biopsies and resected specimens [7]. In the literature, the concordance between the biopsy and final pathology for retroperitoneal sarcoma is in the range of 61–83% [38]. In our study, the concordance between the histology of biopsies and resected specimens was high for malignant tumors (91.2%) and benign tumors (80.6%). The lower percentage for benign tumors is due to the difficulty in differentiating atypical lipomatous tumors from lipomas [39,40]. This is consistent with the results of our study, which showed that the biopsy was inconclusive in six cases because it was not possible to distinguish between atypical lipomatous tumors and lipomas. In a study by Moran et al., tumors with homogeneous fat signals and a maximum size of less than 8 cm were always lipomas (*p* < 0.001) [39].

In accordance with the literature and TARPSWG-, ESMO-, NCCN-guidelines and our study, patients with unclear soft tissue tumors should be discussed with imaging in a multidisciplinary sarcoma board. In the case of superficial, small tumors, a biopsy can be omitted if a complete excisional biopsy is performed and, in the case of lipomatous tumors in the musculoskeletal area that appear benign, if marginal resection is possible. For large intra-abdominal and retroperitoneal tumors, we recommend a biopsy in accordance with the guidelines [2,4,38,41].

Currently, research is being conducted on the development of image recognition through artificial intelligence [42,43,44,45,46,47]. However, there has not yet been a study examining the predictability of tumor entities based on imaging by a sarcoma expert team on an interdisciplinary sarcoma board.

### Limitations

This study had several limitations. First, the number of patients in this study is relatively small. Second, it is a monocentric study. Third, imaging methods varied (MRI, CT, or both), and not all MRI or CT scans were performed according to a standard protocol, as some were conducted in external radiology practices or hospitals. Fourth, the study was designed so that the multidisciplinary sarcoma board only classified tumors as either benign or malignant. However, pathological examination also revealed intermediate tumors, which were excluded from the matching analysis. Fifth, these are real-life data from a team of sarcoma experts, and the benign or malignant prediction was not based on defined tumor criteria.

## 5. Conclusions

In our study, the correlation between cross-sectional imaging of soft tissue tumors and the pathological diagnosis for malignant tumors is significant with a high sensitivity, and for benign tumors significant with a high positive predictive value if demographic (age) and anatomic (site, depth, etc.) factors are considered. However, the study shows that despite the high predictability in an experienced sarcoma center, imaging cannot completely replace biopsies, and caution should be exercised when deciding against a biopsy. It is emphasized that the decision not to perform a biopsy can only be made in cases where lipomatous tumors appear benign on imaging and only in an experienced center. Additionally, further evaluation using radiomics and artificial intelligence is needed to improve the classification accuracy.

## Figures and Tables

**Table 1:** Summary of patients’ characteristics.

	All Patients	Pathological Result			*p*
		Benign	Intermediate	Malignant	
***n***	184	96	17	71	
**Age (years),** **median (range)**	58.5 (21–90)	55 (22–84)	52 (21–80)	66 (29–90)	**<0.001**
**Gender**					0.073
Female Male	94 (51.1%) 90 (48.9%)	54 (56.3%) 42 (43.8%)	11 (64.7%) 6 (35.3%)	29 (40.8%) 42 (59.2%)	
**Localization**					**<0.001**
Extremity	86 (46.7%)	50 (52.1%)	4 (23.5%)	32 (45.1%)	
Intra-abdominal/retroperitoneal	41 (22.3%)	16 (16.7%)	1 (5.9%)	24 (33.8%)	
Trunk	42 (22.8%)	19 (19.8%)	9 (52.9%)	14 (19.7%)	
Head/Neck	15 (8.2%)	11 (11.5%)	3 (17.6%)	1 (1.4%)	
**Imaging**					**<0.001**
MRI	143 (77.7%)	87 (90.6%)	16 (94.1%)	40 (56.3%)	
CT	31 (16.8%)	5 (5.2%)	1 (5.9%)	25 (35.2%)	
MRI and CT	10 (5.4%)	4 (4.2%)	0	6 (8.5%)	
**Pathological grading**					
Malignant tumor, no sarcoma	29 (15.8%)	-	-	-	
G1, sarcoma	6 (3.3%)	-	-	-	
Non-G1, sarcoma	5 (2.7%)	-	-	-	
No existing grading, sarcoma	47 (25.5%)	-	-	-	
Benign tumors	97 (52.7%)	-	-	-	

*p* in bold represents: Statistical significance was set at *p* < 0.05.

**Table 2:** Matching of MDT assessment and pathological result (*p* < 0.001) of benign and malignant tumors without intermediate tumors.

	Interdisciplinary Assessment	
	Benign (*n* = 68; 100%)	Malignant (*n* = 99; 100%)
**Pathological Result** **Benign**	66 (97.1%)	30 (30.3%)
**Malignant**	2 (2.9%)	69 (69.7%)

**Table 3:** Patients’ characteristics of correct and incorrect matching in MDT assessment and pathological results, without intermediate tumors.

	All Patients with Pathological Benign and Malignant Tumor (*n* = 167)			Patients with Benign Evaluation in MDT (*n* = 68)			Patients with Malignant Evaluation in MDT (*n* = 99)		
	Correct Matching	Incorrect Matching	*p*	Correct Matching (Pathological Benign)	Incorrect Matching (Pathological Malignant)	*p*	Correct Matching (Pathological Malignant)	Incorrect Matching (Pathological Benign)	*p*
***n***	135 (100%)	32 (100%)		66 (100%)	2 (100%)		69 (100%)	30 (100%)	
**Age (years),** **median (range)**	60 (22–90)	62 (24–84)	0.499	52.5 (22– 80)	49.5 (37–62)	0.864	66 (29–90)	62.5 (24–84)	0.298
**Gender**			0.698			0.204			0.196
Female Male	66 (48.9%) 69 (51.1%)	17 (53.1%) 15 (46.9%)		37 (56.1%) 29 (43.9%)	0 2 (100%)		29 (42.0%) 40 (58.0%)	17 (56.7%) 13 (43.3%)	
**Localization**			0.266			1.000			0.604
Extremity	63 (46.7%)	19 (59.4%)		32 (48.5%)	1 (50%)		31 (44.9%)	18 (60%)	
Intra-abdominal/ retroperitoneal	32 (23.7%)	8 (25.0%)		8 (12.1%)	0		24 (34.8%)	8 (26.7%)	
Trunk	28 (20.7%)	5 (15.6%)		15 (22.7%)	1 (50%)		13 (18.8%)	4 (13.3%)	
Head/neck	12 (8.9%)	0		11 (16.7%)	0		1 (1.4%)	0	
**Imaging**			0.772			1.000			**0.049**
MRI	101 (74.8%)	26 (81.3%)		63 (95.5%)	2 (100%)		38 (55.1%)	24 (80%)	
CT	26 (19.3%)	4 (12.5%)		1 (1.5%)	0		25 (36.2%)	4 (13.3%)	
MRI and CT	8 (6.7%)	2 (6.3%)		2 (3.0%)	0		6 (8.7%)	2 (6.7%)	
**Grading**			**<0.001**						
Malignant tumor, no sarcoma	29 (21.5%)	0		-	-		-	-	
G1, sarcoma	5 (3.7%)	1 (3.1%)	-	-	-		-	-	
Non-G1, sarcoma	4 (3.0%)	1 (3.1%)	-	-	-		-	-	
No existing grading, sarcoma	30 (22.2%)	0	-	-	-		-	-	
Benign tumors	67 (49.6%)	30 (93.8%)	-	-	-		-	-	

*p* in bold represents: Statistical significance was set at *p* < 0.05.

## Data Availability

The datasets generated during the current study are available from the corresponding author on reasonable request, but are not public due to privacy restrictions, as they were obtained from an interdisciplinary sarcoma board and medical records.

## Supplementary Materials

The following supporting information can be downloaded at: https://www.mdpi.com/article/10.3390/cancers18050784/s1, Table S1: Characteristics of all included patients.

## Abbreviations

The following abbreviation is used in this manuscript:

MDT	Multidisciplinary Tumor Board
TARPSWG	Transatlantic Australasian Retroperitoneal Sarcoma Working Group
ESMO	European Society For Medical Oncology
NCCN	National Comprehensive Cancer Network

## References

[1] WHO Classification of Tumours Editorial Board (2020). Soft Tissue and Bone Tumours.

[2] Gronchi A., Miah A.B., Dei Tos A.P., Abecassis N., Bajpai J., Bauer S., Biagini R., Bielack S., Blay J.Y., Bolle S. (2021). Soft tissue and visceral sarcomas: ESMO-EURACAN-GENTURIS Clinical Practice Guidelines for diagnosis, treatment and follow-up(☆). Ann. Oncol..

[3] Jakob J., Andreou D., Bedke J., Denschlag D., Dürr H.R., Frese S., Gösling T., Graeter T., Grünwald V., Grützmann R. (2023). Ten recommendations for sarcoma surgery: Consensus of the surgical societies based on the German S3 guideline “Adult Soft Tissue Sarcomas”. Langenbeck’s Arch. Surg..

[4] Deutsche Krebsgesellschaft, Deutsche Krebshilfe, AWMF (2022). Leitlinienprogramm Onkologie: S3-Leitlinie Adulte Weichgewebesarkome.

[5] Schmitz F., Sedaghat S. (2024). Inferring malignancy grade of soft tissue sarcomas from magnetic resonance imaging features: A systematic review. Eur. J. Radiol..

[6] Hettler M., Kitz J., Hosseini A.S.A., Guhlich M., Panahi B., Ernst J., Conradi L.C., Ghadimi M., Ströbel P., Jakob J. (2022). Comparing Apparent Diffusion Coefficient and FNCLCC Grading to Improve Pretreatment Grading of Soft Tissue Sarcoma—A Translational Feasibility Study on Fusion Imaging. Cancers.

[7] Johnston E.W., Winfield J.M., Arthur A., Blackledge M., Banerjee U., Basso J., Chowdhury A., Hannay J., Hayes A., Kelly-Morland C. (2025). Robotic MRI/CT Guided Multiregional ‘smart’ Biopsy for Characterization of Tumor Heterogeneity: A Prospective Development Study. Acad. Radiol..

[8] Gennaro N., van der Loo I., Reijers S.J.M., van Boven H., Snaebjornsson P., Bekers E.M., Bodalal Z., Trebeschi S., Schrage Y.M., van der Graaf W.T.A. (2025). Heterogeneity in response to neoadjuvant radiotherapy between soft tissue sarcoma histotypes: Associations between radiology and pathology findings. Eur. Radiol..

[9] Li C., Tan J., Li H., Lei Y., Yang G., Zhang C., Song Y., Wu Y., Bi G. (2025). The value of multiparametric MRI-based habitat imaging for differentiating uterine sarcomas from atypical leiomyomas: A multicentre study. Abdom. Radiol..

[10] Wang B., Guo H., Zhang M., Huang Y., Duan L., Huang C., Xu J., Wang H. (2024). Prediction of soft tissue sarcoma grading using intratumoral habitats and a peritumoral radiomics nomogram: A multi-center preliminary study. Front. Oncol..

[11] Figueiredo G., O’Shea A., Neville G.M., Lee S.I. (2022). Rare Mesenchymal Tumors of the Pelvis: Imaging and Pathologic Correlation. Radiographics.

[12] Sousa F.A.E., Ferreira J., Cunha T.M. (2021). MR Imaging of uterine sarcomas: A comprehensive review with radiologic-pathologic correlation. Abdom. Radiol..

[13] Crombé A., Kind M., Fadli D., Miceli M., Linck P.A., Bianchi G., Sambri A., Spinnato P. (2023). Soft-tissue sarcoma in adults: Imaging appearances, pitfalls and diagnostic algorithms. Diagn. Interv. Imaging.

[14] Blay J.Y., Soibinet P., Penel N., Bompas E., Duffaud F., Stoeckle E., Mir O., Adam J., Chevreau C., Bonvalot S. (2017). Improved survival using specialized multidisciplinary board in sarcoma patients. Ann. Oncol..

[15] Strönisch A., Rau D., Wittenberg S., Kaul D., Koulaxouzidis G., Öllinger R., von Laffert M., Jarosch A., Schäfer F., Keilholz U. (2025). Therapy adherence after interdisciplinary tumour board discussion is associated with improved outcome in soft tissue sarcoma: A Charité Comprehensive Cancer Centre analysis. Int. J. Cancer.

[16] Willner A., Agaimy A., Fechner K., Ott O., Denz A., Weissmann T., Meidenbauer N., Höfler D., Gaipl U., Frey B. (2023). Chemoradiotherapy plus hyperthermia (CRTH) versus chemoradiotherapy (CRT) alone in neoadjuvant treatment of soft tissue sarcoma: Tumor response, treatment toxicity and disease control. Int. J. Hyperth..

[17] Sbaraglia M., Tos A.P.D. (2019). The pathology of soft tissue sarcomas. Radiol. Med..

[18] Schut A.W., Timbergen M.J.M., van Broekhoven D.L.M., van Dalen T., van Houdt W.J., Bonenkamp J.J., Sleijfer S., Grunhagen D.J., Verhoef C. (2023). A Nationwide Prospective Clinical Trial on Active Surveillance in Patients with Non-intraabdominal Desmoid-type Fibromatosis: The GRAFITI Trial. Ann. Surg..

[19] Lazcano C.S., Gronchi A. (2025). Surgical Management of Desmoid Tumors—Patient Selection, Timing, and Approach. Curr. Oncol..

[20] Ressing M., Wardelmann E., Hohenberger P., Jakob J., Kasper B., Emrich K., Eberle A., Blettner M., Zeissig S.R. (2018). Strengthening health data on a rare and heterogeneous disease: Sarcoma incidence and histological subtypes in Germany. BMC Public Health.

[21] Kantzos A.J., Fayad L.M., Abiad J.E., Ahlawat S., Sabharwal S., Vaynrub M., Morris C.D. (2024). The role of imaging in extremity sarcoma surgery. Skelet. Radiol..

[22] Kwee R.M., Kwee T.C. (2022). Diagnostic performance of MRI in detecting locally recurrent soft tissue sarcoma: Systematic review and meta-analysis. Eur. Radiol..

[23] Li X., Wang Q., Dou Y., Zhang Y., Tao J., Yang L., Wang S. (2020). Soft tissue sarcoma: Can dynamic contrast-enhanced (DCE) MRI be used to predict the histological grade?. Skelet. Radiol..

[24] Igrec J., Fuchsjäger M.H. (2021). Imaging of Bone Sarcomas and Soft-Tissue Sarcomas. Rofo.

[25] Noebauer-Huhmann I.M., Vanhoenacker F.M., Vilanova J.C., Tagliafico A.S., Weber M.A., Lalam R.K., Grieser T., Nikodinovska V.V., de Rooy J.W.J., Papakonstantinou O. (2024). Soft tissue tumor imaging in adults: European Society of Musculoskeletal Radiology-Guidelines 2023-overview, and primary local imaging: How and where?. Eur. Radiol..

[26] Tilden W., Saifuddin A. (2021). Telangiectatic soft tissue sarcoma and chronic expanding haematoma: A comparative review of MRI features. Skelet. Radiol..

[27] Bajaj G., Callan A.K., Weinschenk R.C., Chhabra A. (2022). Multiparametric Evaluation of Soft Tissue Sarcoma: Current Perspectives and Future Directions. Semin. Roentgenol..

[28] Shomal Zadeh F., Pooyan A., Alipour E., Hosseini N., Thurlow P.C., Del Grande F., Shafiei M., Chalian M. (2024). Dynamic contrast-enhanced magnetic resonance imaging (DCE-MRI) in differentiation of soft tissue sarcoma from benign lesions: A systematic review of literature. Skelet. Radiol..

[29] Lisson C.S., Lisson C.G., Beer M., Schmidt S.A. (2019). Radiological Diagnosis of Soft Tissue Tumors in Adults: MRI Imaging of Selected Entities Delineating Benign and Malignant Tumors. Rofo.

[30] Lee I.S., Song Y.S., Choi Y.J., Kim J.I., Choi K.U., Kim K., Kang S., Grimm R., Nickel M.D. (2025). Dynamic Contrast-Enhanced MRI in the Evaluation of Soft Tissue Tumors and Tumor-Like Lesions: Technical Principles and Clinical Applications. Korean J. Radiol..

[31] Zhang Y., Yue B., Zhao X., Chen H., Sun L., Zhang X., Hao D. (2020). Benign or Malignant Characterization of Soft-Tissue Tumors by Using Semiquantitative and Quantitative Parameters of Dynamic Contrast-Enhanced Magnetic Resonance Imaging. Can. Assoc. Radiol. J..

[32] Crombé A., Marcellin P.J., Buy X., Stoeckle E., Brouste V., Italiano A., Le Loarer F., Kind M. (2019). Soft-Tissue Sarcomas: Assessment of MRI Features Correlating with Histologic Grade and Patient Outcome. Radiology.

[33] Scalas G., Parmeggiani A., Martella C., Tuzzato G., Bianchi G., Facchini G., Clinca R., Spinnato P. (2021). Magnetic resonance imaging of soft tissue sarcoma: Features related to prognosis. Eur. J. Orthop. Surg. Traumatol..

[34] Bruno F., Arrigoni F., Mariani S., Splendiani A., Di Cesare E., Masciocchi C., Barile A. (2019). Advanced magnetic resonance imaging (MRI) of soft tissue tumors: Techniques and applications. Radiol. Med..

[35] Buchbender C., Heusner T.A., Lauenstein T.C., Bockisch A., Antoch G. (2012). Oncologic PET/MRI, part 2: Bone tumors, soft-tissue tumors, melanoma, and lymphoma. J. Nucl. Med..

[36] Messiou C., Moskovic E., Vanel D., Morosi C., Benchimol R., Strauss D., Miah A., Douis H., van Houdt W., Bonvalot S. (2017). Primary retroperitoneal soft tissue sarcoma: Imaging appearances, pitfalls and diagnostic algorithm. Eur. J. Surg. Oncol..

[37] Swallow C.J., Strauss D.C., Bonvalot S., Rutkowski P., Desai A., Gladdy R.A., Gonzalez R., Gyorki D.E., Fairweather M., van Houdt W.J. (2021). Management of Primary Retroperitoneal Sarcoma (RPS) in the Adult: An Updated Consensus Approach from the Transatlantic Australasian RPS Working Group. Ann. Surg. Oncol..

[38] Purgina B., Di Blasi E., Collini P., Tos A.P.D., Fiore M., Gronchi A., Henderson-Jackson E., Hornick J.L., Mitchell C., Nessim C. (2026). Pathologic Assessment of Retroperitoneal Sarcomas: A Position Paper By the Transatlantic Australasian Retroperitoneal Sarcoma Working Group. Am. J. Surg. Pathol..

[39] Moran L.M., Cai C.Y.L., Ramirez A., Royuela A. (2025). Differentiation of Atypical Lipomatous Tumors from Lipomas: Our Experience with Visual Analysis of Conventional Magnetic Resonance Imaging. J. Imaging.

[40] Natella R., Varriano G., Brunese M.C., Zappia M., Bruno M., Gallo M., Fazioli F., Simonetti I., Granata V., Brunese L. (2023). Increasing differential diagnosis between lipoma and liposarcoma through radiomics: A narrative review. Explor. Target. Antitumor Ther..

[41] von Mehren M., Kane J.M., Agulnik M., Bui M.M., Carr-Ascher J., Choy E., Connelly M., Dry S., Ganjoo K.N., Gonzalez R.J. (2022). Soft Tissue Sarcoma, Version 2.2022, NCCN Clinical Practice Guidelines in Oncology. J. Natl. Compr. Cancer Netw..

[42] Schmitz F., Sedaghat S. (2025). Diagnostic Value of Magnetic Resonance Imaging Radiomics and Machine-learning in Grading Soft Tissue Sarcoma: A Mini-review on the Current State. Acad. Radiol..

[43] Guo J., Li Y.M., Guo H., Hao D.P., Xu J.X., Huang C.C., Han H.W., Hou F., Yang S.F., Cui J.L. (2025). Parallel CNN-Deep Learning Clinical-Imaging Signature for Assessing Pathologic Grade and Prognosis of Soft Tissue Sarcoma Patients. J. Magn. Reson. Imaging.

[44] Schmitz F., Voigtländer H., Strauss D., Schlemmer H.P., Kauczor H.U., Jang H., Sedaghat S. (2024). Differentiating low- and high-proliferative soft tissue sarcomas using conventional imaging features and radiomics on MRI. BMC Cancer.

[45] Benhabib H., Brandenberger D., Lajkosz K., Demicco E.G., Tsoi K.M., Wunder J.S., Ferguson P.C., Griffin A.M., Naraghi A., Haider M.A. (2025). MRI Radiomics Analysis in the Diagnostic Differentiation of Malignant Soft Tissue Myxoid Sarcomas From Benign Soft Tissue Musculoskeletal Myxomas. J. Magn. Reson. Imaging.

[46] Voigtländer H., Kauczor H.U., Sedaghat S. (2025). Diagnostic utility of MRI-based convolutional neural networks in soft tissue sarcomas: A mini-review. Front. Oncol..

[47] Crombé A., Matcuk G.R., Fadli D., Sambri A., Patel D.B., Paioli A., Kind M., Spinnato P. (2023). Role of Imaging in Initial Prognostication of Locally Advanced Soft Tissue Sarcomas. Acad. Radiol..

